# Endocarditis Presenting as Right Atrial Mass, Diagnosed with 18F-Fluorodeoxyglucose-PET: A Case Report

**DOI:** 10.14797/mdcvj.1223

**Published:** 2023-04-24

**Authors:** Alexandra Ramos, Isadora Sande Mathias, Mouaz Al-Mallah, Miguel Quinones

**Affiliations:** 1 School of Engineering Medicine, Texas A&M University, Bryan, Texas, US; 2 Houston Methodist DeBakey Heart & Vascular Center, Houston, Texas, US

**Keywords:** endocarditis, FDG-PET, transesophageal echocardiogram

## Abstract

We report a case of a 55-year-old male with a history of methicillin-resistant staphylococcus aureus bacteremia whose initial transesophageal echocardiography revealed a cardiac mass attached to the right atrium. Because of the uncommon location of the mass, 18F-fluorodeoxyglucose-PET was used to confirm the diagnosis of infective endocarditis.

## Introduction

The incidence of infective endocarditis (IE) has steadily increased by 11 to 15 cases per 100,000 people per year between 2000 and 2011.^1^ Staphylococcus aureus is the most common bacterial cause of these IE cases, accounting for 60% to 90%, particularly right-sided IE. The complications of right-sided IE include those that affect the heart valves, such as regurgitation and annulus abscess formation, as well as peripheral effects including septic shock and septic emboli.

If the clinical presentation is suspicious for IE based on the Duke criteria, then blood cultures and imaging can be performed to confirm the diagnosis and guide treatment. The initial imaging choice of a cardiac mass with unclear etiology may include transthoracic echocardiography (TTE) since it is quick and noninvasive. Other studies may be selected based on initial echocardiogram results, but they include transesophageal echocardiography (TEE), cardiac magnetic resonance imaging (MRI), computed tomography (CT), and positron emission tomography (PET). TEE may be able to provide more precise information about the anatomic location of the cardiac mass, particularly if it is located in the atria, and changes to the surrounding tissues. While PET is more commonly used in the diagnosis of cardiac masses of malignant etiology, it also has been used to diagnose IE in prosthetic valves, specifically, because it reflects the metabolic activity of the mass. We discuss a case of endocarditis presenting as a right atrial mass on TEE, where PET was fundamental to confirm the diagnosis.

## Case Presentation

A 55-year-old male presented to the emergency department (ED) with left arm/hand pain, associated with swelling and erythema, after falling on an outstretched arm the previous week. The patient reported having a fever a few days prior to his ED admission. His past medical history is significant for coronary artery disease, stroke, and end-stage renal disease on hemodialysis through left femoral tunneled line due to central venous stenosis. He had multiple dialysis line exchanges in the setting of polymicrobial line infections, and the last cultures had cleared 4 months prior.

On physical exam, the patient was ill-appearing. The affected forearm and hand had swelling and tenderness. The wrist had decreased range of motion with a normal pulse. The left femoral dialysis catheter had mucoid drainage without erythema. The patient was febrile, with a temperature of 101. His heart rate was 88 bpm, blood pressure was 128/65 mm Hg, SpO2 was 98%, and respiratory rate was 18 breaths per minute.

Initial laboratory studies showed elevated white blood cell (WBC) with neutrophilic predominance and troponin T of 154 (lab reference range of 0-19 ng/L). Blood cultures were positive for methicillin-resistant staphylococcus aureus (MRSA) in two bottles.

A chest x-ray taken on admission showed a normal cardiomediastinal silhouette.

An MRI of the left wrist was performed and showed distal radial-ulnar joint and pancarpal joint effusions with diffuse soft tissue edema. Ultrasound-guided aspiration of the left wrist was performed and aspirate had negative growth on cultures after 4 days.

**Figure 1:**
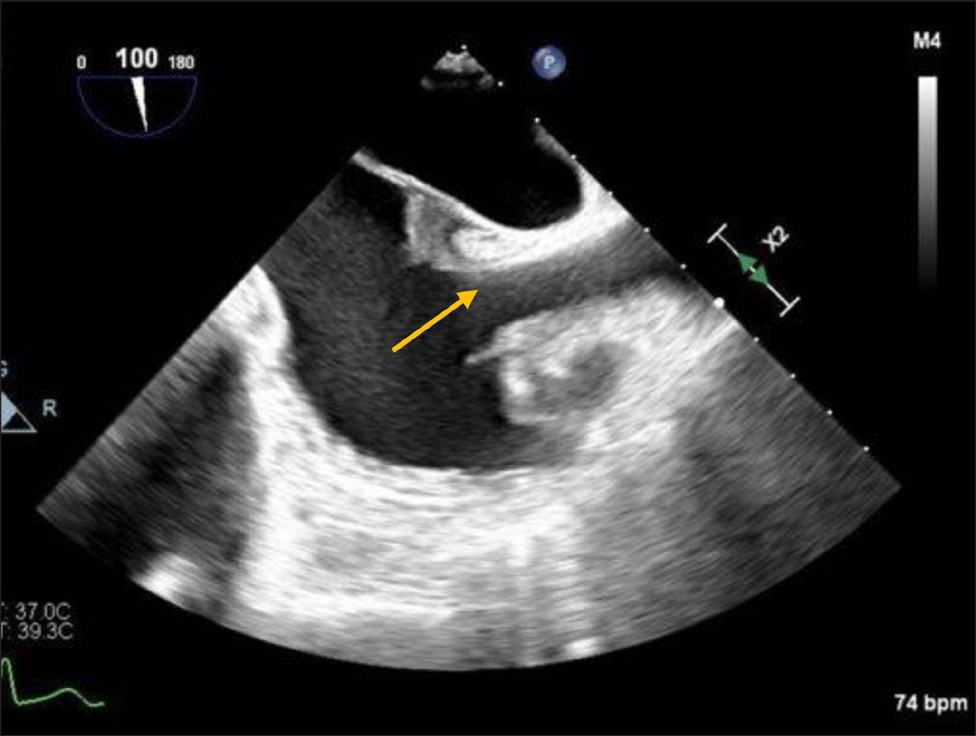
Cardiac mass in the atrial septum at the right atrial-superior vena cava junction (arrow).

**Figure 2:**
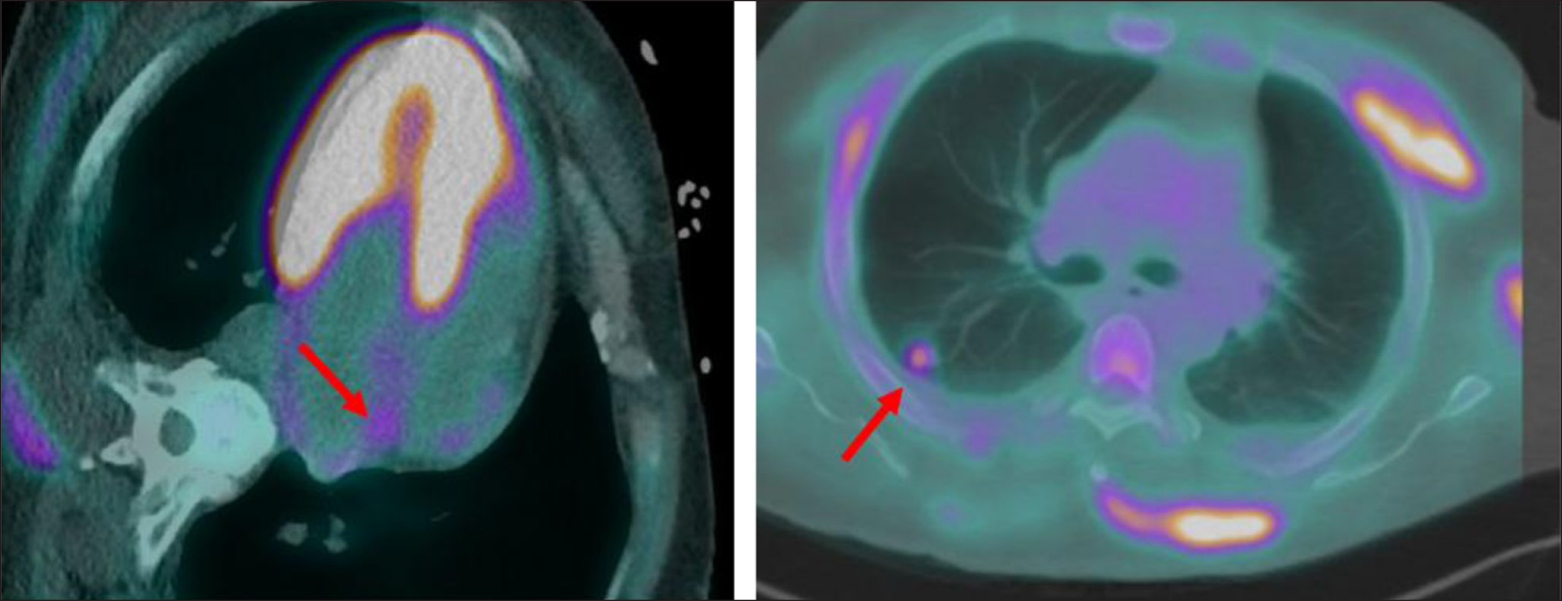
Increased 18F-fluorodeoxyglucose uptake in the interatrial septum and evidence of septic emboli in the right lung (arrows).

Due to the patient’s history of recurrent line infections and recurrent MRSA bacteremia, the left femoral line was removed and a TEE was ordered. The TEE showed a 1.5-cm partially mobile echodensity attached to the interatrial septum, as shown in Figure 1 and Videos 1 and 2, but it was unclear if the mass was a vegetation or another etiology such as a thrombus or tumor. Agitated saline contrast study showed right to left shunting through a patent foramen ovale (Video 3). 18F-fluorodeoxyglucose-PET (FDG-PET) was recommended to differentiate the etiology of the mass. There was evidence of FDG uptake in the right atrium near the right atrial-superior vena cava junction (as shown in Figure 2) consistent with infectious etiology. In addition, there was evidence of septic embolism and multiple inflammatory lung nodules.

**Video 1:** Transesophageal echocardiogram showing right atrial septal mass at the right atrial-superior vena cava junction. Four chamber view; see also at https://youtu.be/nFFyvnVpHPc.

**Video 2:** Transesophageal echocardiogram showing right atrial septal mass at the right atrial-superior vena cava junction. Bicaval view; see also at https://youtu.be/yCnJp7fGYeM.

**Video 3:** Agitated saline contrast showing a patent foramen ovale; see also at https://youtu.be/AehcLTbyECs.

The patient was started on intravenous antibiotics and a new line was placed a few days later. After adequate treatment and culture-negative studies, the patient was discharged and continues to do well.

## Discussion

In the present case, a 55-year-old male with recurrent catheter-associated bloodstream infections and ongoing hemodialysis presented with fevers and drainage from his dialysis access site. A mass attached to the interatrial septum initially identified by TEE was confirmed to be infectious in origin, as opposed to a thrombus, through the use of FDG-PET.

To diagnose infective endocarditis, multiple blood cultures, transthoracic or transesophageal echogram, and the Duke Criteria are used, frequently in combination. In the case of this patient, a TEE showed a mass with limited tissue characterization. Infective endocarditis rarely presents as a mass in one of the cardiac chambers, as in this case, which warranted further investigation with FDG-PET.

Previous literature has supported the use of FDG-PET as a diagnostic tool for infective endocarditis, although it is more commonly used to evaluate infective endocarditis of prosthetic valves.^2^,^3^ In a meta-analysis of 13 studies involving 537 patients, the pooled sensitivity for the diagnosis of infective endocarditis using FDG-PET was 76.8% and pooled specificity was 77.9%.^4^ Diagnostic accuracy in prosthetic valves was slightly higher in sensitivity but reduced in specificity. Furthermore, recent analyses of FDG-PET as a diagnostic tool for IE have shown increased accuracy.^4^,^5^,^6^

FDG-PET is not frequently used to identify and diagnose infective endocarditis in patients without preexisting prosthetic cardiac material. In one case report, FDG-PET was used to diagnose endarteritis within a case of coarctation of the aorta without any prosthetic material.^7^ Infected intracardiac mass is a rare presentation of IE. In one case report, infective endocarditis caused by MRSA was diagnosed using TEE and identified as a pedunculated mass on the posterior wall of the left atrium.^8^ In this case, blood cultures positive for MRSA prompted the start of vancomycin; however, the mass was not found until 1 week later when the patient’s symptoms had not improved. This is similar to the clinical presentation and observations made in this case report, although FDG-PET was not used as part of the diagnosis.

## Conclusion

This is a case of MRSA infective endocarditis resulting in a large mass-like lesion within the right atrium identified via FDG-PET as infectious in origin. This approach reflects the growing diagnostic value and accuracy of FDG-PET in non-prosthetic intracardiac assessment of endocarditis.
